# Assessing Health-Related Quality of Life, Morbidity, and Survival Status for Individuals With Down Syndrome in Pakistan (DS-Pak): Protocol for a Web-Based Collaborative Registry

**DOI:** 10.2196/24901

**Published:** 2021-06-03

**Authors:** Ayat Siddiqui, Laila Akbar Ladak, Abdul Momin Kazi, Sidra Kaleem, Fizza Akbar, Salman Kirmani

**Affiliations:** 1 Department of Pediatrics Aga Khan University Hospital Karachi Pakistan; 2 Faculty of Medicine and Health Susan Wakil School of Nursing and Midwifery Sydney Nursing School, The University of Sydney Sydney Australia

**Keywords:** Down syndrome, registry, web-based registry, health-related quality of life, lower-middle income country, mobile health, patient-reported outcome

## Abstract

**Background:**

Down syndrome is the most common chromosomal disorder, with a global incidence of 1 in 700 live births. However, the true prevalence, associated morbidities, and health-related quality of life (HRQOL) of these individuals and their families are not well documented, especially in low- and middle-income countries such as Pakistan. Disease-specific documentation in the form of a collaborative registry is required to better understand this condition and the associated health outcomes. This protocol paper describes the aims and processes for developing the first comprehensive, web-based collaborative registry for Down syndrome in a Pakistani cohort.

**Objective:**

This study aims to assess the HRQOL, long-term survival, and morbidity of individuals with Down syndrome by using a web-based collaborative registry.

**Methods:**

The registry data collection will be conducted at the Aga Khan University Hospital and at the Karachi Down Syndrome Program. Data will be collected by in-person interviews or virtually via telephone or video interviews. Participants of any age and sex with Down syndrome (trisomy 21) will be recruited. After receiving informed consent and assent, a series of tablet-based questionnaires will be administered. The questionnaires aim to assess the sociodemographic background, clinical status, and HRQOL of the participants and their families. Data will be uploaded to a secure cloud server to allow for real-time access to participant responses by the clinicians to plan prompt interventions. Patient safety and confidentiality will be maintained by using multilayer encryption and unique coded patient identifiers. The collected data will be analyzed using IBM SPSS Statistics for Windows, Version 22.0 (IBM Corporation), with the mean and SD of continuous variables being reported. Categorical variables will be analyzed with their percentages being reported and with a *P* value cutoff of .05. Multivariate regression analysis will be conducted to identify predictors related to the HRQOL in patients with Down syndrome. Survival analysis will be reported using the Kaplan-Meier survival curves.

**Results:**

The web-based questionnaire is currently being finalized before the commencement of pilot testing. This project has not received funding at the moment (ethical review committee approval reference ID: 2020-3582-11145).

**Conclusions:**

This registry will allow for a comprehensive understanding of Down syndrome in low- and middle-income countries. This can provide the opportunity for data-informed interventions, which are tailored to the specific needs of this patient population and their families. Although this web-based registry is a proof of concept, it has the potential to be expanded to national, regional, and international levels.

**International Registered Report Identifier (IRRID):**

PRR1-10.2196/24901

## Introduction

### Down Syndrome

Down syndrome, a chromosomal disorder, is caused by the presence of a complete or partial third copy of chromosome 21 [1]. Owing to the presence of extrachromosomal material, there is an amplified effect on some genes, which leads to the classic features and associated medical conditions of Down syndrome. Patients with Down syndrome commonly present with characteristic facial features, delayed development, and intellectual disability [2-4]. Down syndrome is the most common genetic cause of intellectual disability [5], with patients experiencing cognitive impairment and difficulty in learning [6]. Medically, patients are affected with various conditions such as obstructive sleep apnea, congenital heart defects, and leukemia, among others [7].

Down syndrome is prevalent in all populations, races, and ethnicities. The Center for Disease Control reported that Down syndrome is the most common chromosomal disorder, with an incidence of 1 in 700 births [8]. However, data regarding the incidence and prevalence of Down syndrome for low- and middle-income countries (LMICs) are scant. According to one study, 1 in 300 babies in Pakistan are diagnosed with Down syndrome [9]. However, for a national prevalence rate, more studies are required to compare the incidence rate in an LMIC against high-income countries.

### Health-Related Quality of Life in Patients With Down Syndrome

Health-related quality of life (HRQOL) is an important parameter to assess as routine clinical assessments may not reflect the treatment burden, functional limitations, or the need for adjustment to disability that a person may experience. HRQOL assessment is complex as it requires understanding an individual’s perception, experiences of their health-related issues, and perceived level of adaptation and coping.

As Down syndrome is a chronic condition, the impact of its related comorbidities is often lifelong and debilitating. Poor HRQOL has been reported in patients with Down syndrome with regard to physical, emotional, social, psychosocial, and psychological well-being [10-12]. Although this is often overlooked, it is important to acknowledge that Down syndrome affects the patient’s whole family in many aspects, particularly financially and emotionally, as additional responsibilities are placed on the caregivers [13]. Multiple sociocultural factors can influence caregivers’ experiences and perceptions of the patient’s condition. For instance, a study conducted in Pakistan reported that parents perceived Down syndrome as a consequence of their deeds and experienced feelings of guilt regarding it [14,15]. Furthermore, a lack of understanding about the disease and its management added additional stress to these parents and hampered their ability to provide care to their child [16,17]. Early intervention, vocational therapy, and medical management are associated with improved functioning in children in terms of health and social outcomes [18,19]. Unfortunately, delayed diagnosis and management are common in LMICs, with some children being diagnosed as late as age 7 [14,15]. Owing to the various region-specific difficulties, results from studies regarding HRQOL in this population would vary from those carried out in a resource-rich setting [13].

### Challenges of Down Syndrome in LMICs

The challenges faced by LMICs regarding health care delivery are unique and multifactorial. The obstacles include a lack of resources, inadequate skills and training, nonstandardized documentation, a lack of quality check frameworks, a lack of health insurance (with patients predominantly being forced to self-pay), and a lack of system infrastructure and support. Patients and their caregivers often do not feel that they have the power to verbalize what is important to them as access to any available health care becomes the predominant concern for a family. Furthermore, because of a lack of resources, there is a prevailing problem of the delayed diagnosis of Down syndrome. Pakistan, being an LMIC, has disparities in terms of health care provision. There are health care centers where antenatal monitoring is at par with developed countries; however, it is not uniformly available in the sixth most populous country. To facilitate early diagnosis, hospitals can and have started connecting with physicians and pediatricians who can enable the early diagnosis of Down syndrome. Unfortunately, no registries or formal collaborations exist to highlight these unique issues, risks, and comorbidities in Down syndrome, particularly in Pakistan.

### Rationale

Disease-specific documentation in the form of a collaborative registry is crucial and serves an important purpose of understanding the disease and its associated health outcomes better. Patient-reported outcomes and patient-related experience measures have been noted as significant parameters for the implementation of the philosophy of *Value-Based Health Care*. This will be the first comprehensive registry for Down syndrome and its associated health problems in a Pakistani cohort. The information regarding individuals with Down syndrome and their families can be documented and will allow measurement of the impact of early health care interventions and routine medical follow-up on the well-being of individuals with Down syndrome and their families. Subsequently, through this registry, we can further investigate the factors affecting health care outcomes in a large cohort. This will lead to further research to improve interventions, offer novel treatment, and improve the quality of care for people with Down syndrome. To date, there are limited published data about the determinants of the well-being of young individuals with a diagnosis of Down syndrome, particularly in LMICs. Therefore, it is important to investigate the determinants and the associated protective factors and risk factors affecting HRQOL in individuals with Down syndrome and their families.

### Aim

This web-based collaborative registry aims to identify the long-term survival, morbidity outcomes, and HRQOL and the experiences in patients with Down syndrome.

### Objectives

The main objectives of this study are (1) to assess the long-term survival, morbidity outcomes, and effects on HRQOL in patients with Down syndrome in Pakistan and (2) to determine predictors of HRQOL in patients with Down syndrome in Pakistan.

## Methods

### Study Site and Participants

This will be a prospective registry that will be conducted at the outpatient clinic of Aga Khan University Hospital (AKUH) and the Karachi Down Syndrome Program (KDSP). Patients of any age and sex with Down syndrome and their families visiting AKUH and the KDSP will be approached and invited to participate in this collaborative registry. After giving their informed consent and assent, patients will be interviewed in person or remotely via a telephone call, a Zoom meeting, or WhatsApp, as applicable. As the patients are being interviewed, the questionnaire responses will be recorded on a smart device controlled by the research assistant. According to the American Academy of Pediatrics, the health supervision of children with Down syndrome is categorized according to age [7] (Textbox 1).

**American Academy of Pediatrics health supervision schedule.**

**Birth to 1 month**

- Evaluation for heart defects, feeding problems, cataracts at birth, duodenal atresia, congenital hearing loss, constipation, gastroesophageal reflux, congenital hypothyroidism, hematological problems, and apnea.

**1 month to 1 year**

- Previous reports reviewed and concerns addressed (existing and new).
- Tympanometry may be ordered for detection of middle ear disease.
- 0-5 months: Ophthalmology referral to evaluate nystagmus, cataracts, and strabismus [17].
- 6 and 12 months: Thyroid function and cardiac screenings.
- Signs of neurological dysfunction or seizure.

**1-5 years**

- Monitor growth and development of child.
- Repeat screening for hearing impairment every 6 months and vision evaluation.
- Further evaluation for specific conditions as relevant.
- Impact of early intervention (speech, occupational, and physical therapies) reviewed.

**5-13 years**

- Annual visits with similar monitoring of growth and development.
- Specific attention given to evaluating age-specific BMI and maintaining healthy diet to prevent obesity.
- Evaluate social function and behavioral development of the child at this stage, both at home and during school.

This routine health care surveillance is crucial for improved HRQOL of the patients and for the well-being of their families [19]. We will follow up with participants in accordance with this schedule and then schedule routine follow-ups into adulthood to assess the patients’ quality of life and health status. The Pediatric Quality of Life (PedsQL) questionnaire forms have been used successfully in previous studies in Pakistan, and the patients were followed up into adulthood [20].

### Web-Based Collaborative Registry

#### Overview

A web-based registry will be developed for data entry on the portal, with the data being stored on a cloud server. Data entry will occur in real time by the research assistant, and access to the data will be restricted to relevant study team members, physicians, or individuals involved in the care of the patient through a login ID and password. After each data entry, a PDF report will be generated, which can be printed and added to patients’ medical records and viewed by the physician in real time. The collected data can provide direction for addressing the needs of the patients with Down syndrome and their families. The main registry database will reside on a central computer at AKUH and will be managed by the study staff. A web-based dashboard will be designed to report the registry progress. All study staff will undergo basic research ethics training. Participants’ information will be given a unique patient identifier code, and no personal information will be shared.

#### Mobile Health Digital Platform and Data Collection

The process of gathering information (variables of interest) will be executed in a systematic and digitized manner. All data will be collected on smart devices (tablets, mobile phones, etc). The data collection apps for the electronic questionnaire will be developed using the Android Software Development Kit or Open Data Kit, an open-source software package, and utilize in-house front-end software for retrievable variables. The electronic questionnaires will contain skip patterns to navigate the user to relevant questions. Electronic case report forms will also be loaded with valid values and specifications for valid ranges and range checks. It will also automatically record the start and end times of each interview, which will help monitor and manage the interview protocol. The collected data will be stored on the smart device and will be synchronized to AKUH data servers through http secure. The information from the electronic questionnaire will be uploaded to the central server for close to real-time monitoring using the dashboard interface.

#### Data Management

The clinical staff, under the supervision of the principal investigator and coinvestigators, will be responsible for collecting the data. The clinical team collecting the data will review them for completeness, accuracy, and visualization. The study manager or coordinator will provide guidance to the study staff on the corrections and edits that may be needed in the electronic data. The registry data manager will be responsible for the overall data management and data quality assurance. Once the data are transferred to the central registry database, the data management team will ensure completeness and validity. Only the data management staff will be authorized to make modifications to the registry database in consultation with the program manager or study coordinator with documentation of specific reasons. Identifiers will only be visible to the authorized study staff.

There are 2 unique identifiers: medical record number and registry ID number. The registry administrator and principal investigator of the study will assign access to the study according to the portfolio of the study staff and investigators. For example, physicians will have full access to clinical data and limited access to research data and vice versa for research staff. However, the principal investigator and nominated investigators of the study will have full access to the clinical and research data.

#### Data Security

All the devices used for collecting data will be password protected. Only designated personnel will have access to these data for monitoring and operational purposes. All data collected on handheld devices as well as during transmission will be encrypted using the Rivest-Shamir-Adleman encryption. Weekly data backup will be scheduled on a different machine, and off-site backups will occur at regular intervals. This web-based registry is an electronic portal consisting of multiple dashboards to keep track of the registry database in real time and visualize the progress of the study at a glance. It will be hosted on a Google Cloud server with multiple layers of encryption to ensure rigorous security practices against threats to the infrastructure from both internal and external factors. Access to sensitive data will be protected by advanced tools, whereas database and file storage will be protected by AES256 or AES128 encryption. This will help in guarding against unauthorized access and service interruptions.

#### Recruitment Process

Eligible patients with Down syndrome and their parents or guardians visiting the genetics clinic at AKUH and at the KDSP will be approached to participate in the registry. AKUH is one of the only Joint Commission International–certified tertiary care centers in Pakistan. Patients from all over the country and the international community come to receive state-of-the-art care at this leading hospital. The KDSP is a nonprofit organization formed by parents and passionate individuals interested in improving the lives of children with Down syndrome, with a focus on occupational therapy and improving their quality of life [21]. This collaboration allows an insight into not only the health care perspective but also the child’s community and social support. In addition, patients who are not scheduled for a clinic visit will be approached through a telephone call, a Zoom meeting, or WhatsApp to minimize patient exposure in the light of the COVID-19 pandemic. The participants will be provided with information related to the registry. An interview slot will be allocated to interested eligible patients and their parents to schedule their interviews. Informed consent will be obtained from patients and their [22] parents both verbally and in writing when applicable, with a witness present when consent is obtained remotely, before the commencement of the interview. Participants will be offered a choice to receive that information in English or Urdu, the latter being the nationally spoken language of Pakistan. Assent will also be obtained from patients aged 7 to 18 years, with a witness being present. A copy of the informed consent and assent will be provided to the participants. The interviews will consist of a series of tablet-based questionnaires. The interviews will be conducted at the KDSP, in a designated room in the clinical trials unit at the AKUH, or remotely. When conducted remotely, the research assistant will record the participant responses on an electronic questionnaire, which will be automatically saved to the web-based registry platform.

#### Data Collection

Baseline surveys regarding the sociodemographic variables, clinical history, outcomes, and health information of the patient will be administered to the patients or their caregivers by a research assistant. The baseline survey form has been developed based on the intake form used by clinicians and researchers at the National Institute of Health’s Down Syndrome Registry, which is publicly available through DS-Connect [23], and Children’s Hospital of Boston Down Syndrome clinic, which provided the information after an email request. The questions have been screened and modified by experts in the fields of genetics and pediatric development. Trained staff will conduct the interviews using a tablet. Business rules, consistency checks, and skips will be incorporated, and important fields will be marked as a must to enter.

The PedsQL generic core (physical, emotional, social, psychosocial, and school or work domains), PedsQL Cognitive scale (cognitive functioning), and PedsQL general well-being questionnaires will be used to explore HRQOL. These questionnaires have been widely used in other settings to explore HRQOL [24]. The PedsQL Family Impact Module (physical, emotional, social, cognitive, communication, concerns related to the child, daily activities, and family relationship) will be used to explore the impact of Down syndrome on the family as a unit. In addition, the PedsQL Health Satisfaction Generic Module (information, family inclusion, communication, technical skills, emotional needs, and overall satisfaction) will be explored by the parents. All these questionnaires have well-established validities and reliabilities [25]. They are available for populations of different ages as well as their parents or proxies for young children (5-7 years), children (8-12 years), teenagers (13-18 years), young adults (18-25 years), and adults (>26 years). There is also a guardian or parent version only for toddlers (2-4 years), given the developmental limitations on self-reporting for children younger than 5 years.

**Figure 1:**
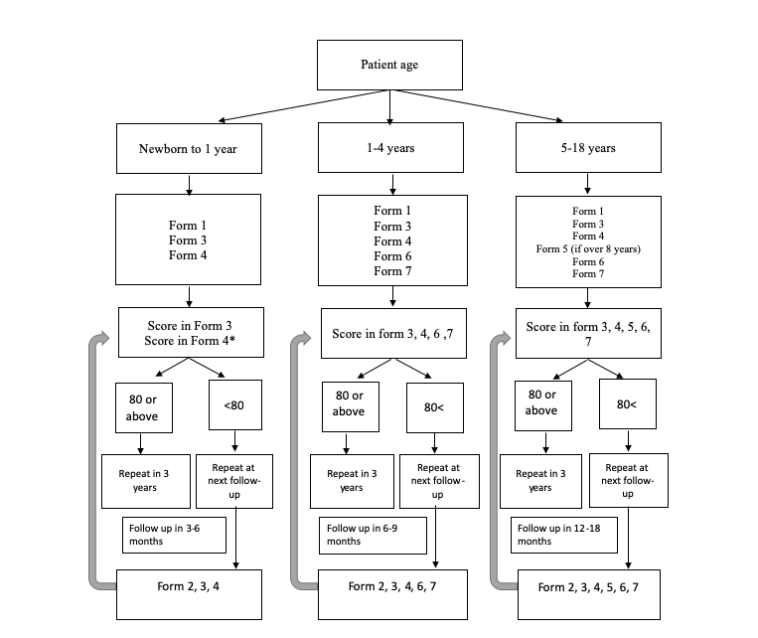
Web-based registry questionnaire administration algorithm. Forms attached—Form 1: initial baseline intake form; Form 2: follow-up intake form; Form 3: family report; Form 4: health care satisfaction; Form 5: general well-being; Form 6: cognitive functioning scale; and Form 7: Pediatric Quality of Life inventory. *Assess score of each form independently and base decision of administering form again at follow-up appointment on cutoff scores.

All the age-relevant questionnaires will be administered when the participants are enrolled at their initial interview. At the conclusion of a questionnaire pertaining to HRQOL, a score is generated. On the basis of a preset cutoff, the registry algorithm will alert the interviewer at the next follow-up that a particular questionnaire needs to be re-administered. If the participant’s score is above the cutoff of 80, the algorithm will inform the interviewer of the need to repeat the particular questionnaire in 2 to 3 years. The scores of each questionnaire will be assessed independently. The follow-up timing of each patient depends on their age cohort (Figure 1). Follow-up appointments can continue into adulthood as there are specific PedsQL questionnaires designed for adult participants. Given the frequent follow-up appointments, financial constraints on patients and their families must be considered in a resource-constrained setting. In Pakistan, AKUH along with other nongovernment organizations are offering web-based consultations on subsidized clinical charges. Even when full charges are being received, web-based consultations allow families to save on the cost of traveling. To make it sustainable, our children’s hospital has pediatric welfare funds that can help ease the financial burden.

**Figure 2:**
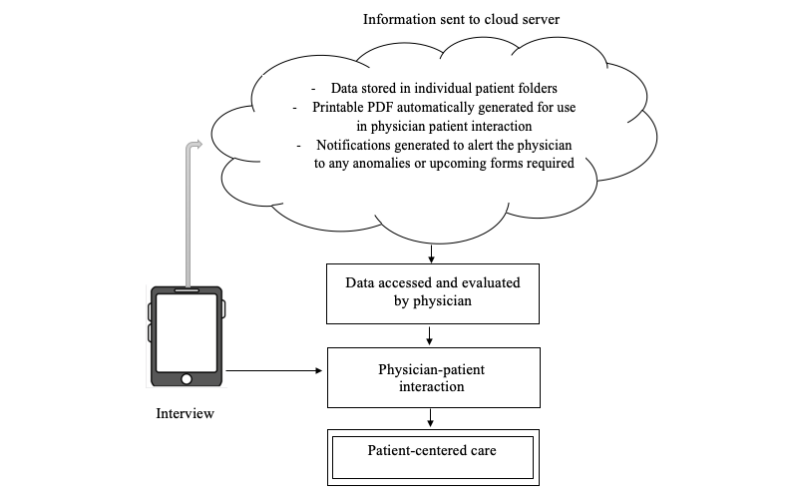
Web-based registry data pathway.

Data will be collected in real time using a tablet-based questionnaire. The data will be collected from the patient (≥5 years), their parents or guardians (for patients aged 2-18 years), and from patient files as applicable. Once collected, the data will be uploaded to the cloud server and stored in the patient’s individual folder based on their unique identification number. The stored data can be summarized in the form of an automatically generated PDF to aid with the following physician-patient interaction, and notifications regarding anomalies or upcoming forms required will be alerted (Figure 2). Details of the variables, their sources, and the questionnaires used, including sociodemographic variables, family history, birth history, immunization history, developmental history, medical history, comorbidities, medications, physical examination, health care utilization, functional status, and impact on family, are given in Table 1.

Permission to use the PedsQL HRQOL questionnaires has been obtained. These questionnaires were originally in English and have been translated to Urdu and then back-translated by an expert panel. Any discrepancies with the translation will be reported. The form will then be pretested for content and face validities. The final version will then be submitted to Mapi Research (PedsQL parent organization) along with the whole translation report. Differences in the Pakistani population are anticipated, and modifications to the PedsQL forms can be suggested to the parent organization following pilot testing. For patients who were already being treated at AKUH and the KDSP, the clinical notes on the file can be used retrospectively to gather more data regarding the participant.

**Table 1:** Variables included in quality-of-life assessment using self-developed form as a data collection tool.

Domains and domain aspects			Data source
**Sociodemographic variables**			
	Age	Patient, parents, sibling	
	Education	Patient, parents, sibling	
	Family income	Patient, parents, sibling	
	Family structure, number of members	Patient, parents, sibling	
	Ethnicity	Patient, parents, sibling	
	City of residence	Patient, parents, sibling	
**Family history**			
	Consanguineous marriage	Parents	
	Sibling details	Parents	
	History of any chromosomal abnormality or disability	Parents	
**Birth history**			
	Anthropometrics, parental age, delivery details etc	Parents	
**Immunization history**			
	Birth till current immunization as per EPI^a^	Parents, immunization card	
**Developmental history**			
	Walking, feeding, speech, etc	Parents	
**Medical history of patient (Down syndrome)**			
	Down syndrome diagnosis history, chromosomal tests done, antenatal, or postnatal monitoring etc	Parents, laboratory reports	
**Comorbidities**			
	Other medical problems	Parents	
**Medications**			
	Surrogate for health status	Parents	
**Physical examination**			
	Gastrointestinal, CVS^b^, CNS^c^, musculoskeletal, skin, genitourinary, allergies	Physician or medical record file	
**Health care utilization**			
	Number of hospitalizations, surgical procedures, number of hospital visits, interventions, therapy, services utilized, cost of each therapy and visit, payment mode (self, insurance, philanthropist, hospital finance assistance, etc)	Parents	
**Functional status**			
	Physical, emotional, social, and psychosocial status and school or work	Patient or parent	
	Cognition	Patient or parent	
	General well-being	Patient or parent	
**Impact on the family**			
	Parental QOL^d^	Parents	
	Family functioning	Parents	
	Satisfaction with the health care received or receiving	Parents	

^a^EPI: Expanded Program on Immunization.

^b^CVS: cardiovascular system.

^c^CNS: central nervous system.

^d^QOL: quality of life.

#### Data Analysis

The data will be exported to SPSS version 22 (IBM Corporation) for analysis. For descriptive analysis, the number of participants enrolled, mean and SD of continuous variables, and percentages for the categorical variables will be reported.

The HRQOL and health care utilization questionnaires have very similar response items and scoring. These questionnaires will capture patients’ experiences in the preceding month. Both patients and parents will be asked to rate their HRQOL from 0 to 4, with 0 being “never a problem” and 4 being “almost always a problem.” Responses will be reverse scored and linearly transformed to 0-100, with higher scores indicating better HRQOL. If more than 50% of the items in the scale are missing, the scale scores will be considered as missing data. The mean score of the individual domains will be calculated by adding the sum of the items to the number of items answered. The total score will be the sum of all the items over the number of items answered on all scales.

The registry will also identify the predictors of HRQOL in the patients with Down syndrome. For all predictor variables (sociodemographic and clinical parameters), the univariate analysis will be performed with the HRQOL domains. Studies have shown that in older patients with Down syndrome, poor HRQOL was reported because of health problems, limited social relationships, and restricted educational and employment opportunities [12,26]. However, although there are data regarding parent-reported HRQOL [27] and even HRQOL of parents of children with Down syndrome [27], there are very limited data regarding self-reported HRQOL in children with Down syndrome. Each predictor variable will be regressed against the HRQOL variable to assess the eligibility of a predictor variable to be included in the final model. The criteria for inclusion of a predictor variable will be set at a *P* value cutoff of .25 at the univariate analysis level. Insignificant variables that do not meet the cutoff criterion will be removed from the model at the univariate analysis level. The significant variables will then be assessed for multicollinearity. In the multivariable regression analysis stage, the cutoff for the *P* value will be set at .05. Variables will be added to the model until the overall model remains significant through the manual stepwise model-building technique. After the multivariable analysis, the interaction will be assessed among the predictor variables. The cutoff at this stage will be set at .1. For a predictor variable to have an interactive effect with another variable, the *P* value of the respective variables should be less than or equal to the set cutoff, that is, .1. Observations of the significant variables will also be vetted for outliers.

The Kaplan-Meier survival curves will be used to report survival status. Survival will be analyzed for the cohort with respect to variables such as age, sex, and comorbidities, with *P*<.05 being accepted as statistically significant.

### Ethical Considerations

The registry has received approval from the ethics review committee at AKUH, Pakistan. Informed consent will be obtained from patients and their parents both orally and in writing, and participants will be offered a choice to receive that information in English or Urdu. Assent will also be obtained from patients aged 5 to 18 years. The study participants will have a unique identifier to maintain patient confidentiality. Original and backup files will be archived in password-protected computers or servers at AKUH. Data confidentiality will be maintained at all times. No personal identifiers will be used in any reports or publications of this study. No individual identifiers such as names of participants or areas of location will be shared. Although the risks associated with this study are negligible, it is possible that participants may become distressed while telling their story. In this instance, we will follow the participant distress protocol developed for this study. Data confidentiality and archiving will be maintained according to the Good Clinical Practice Guidelines. Data findings will be disseminated through peer-reviewed publications and presentations at national and international conferences.

## Results

This study was approved by the AKUH Ethics Review Committee (Reference ID: 2020-3582-11145). Currently, no funding is available, but opportunities are being explored. Furthermore, we are preparing to begin pilot testing of the registry by September 2021.

## Discussion

### Benefits of a Patient Registry

In recent years, the utilization of technology has profoundly impacted and benefited patient care. The dearth of information available at a moment’s notice allows the practice of evidence-based medicine and provides the ability to monitor trends and patterns. A significant source of this information is from biobanks and data registries, and with the use of technology and web-based designs, these data can be accessed in real time. The true beneficiaries of data registries are the patients as the increased information allows for patient-centered care and real-time decisions that can impact patient lives [28]. A majority of registries are formed and operated in high-income countries, where there is the presence of established health care systems to ease patients with respect to costs and follow-up appointments and the invaluable use of electronic health records. However, although they face unique challenges and obstacles [29], a number of registries are being successfully operated in LMICs [30,31], and the data obtained have a great impact on patient care and further research in a setting where there is a lack of availability of organized data.

### Limitations

At its conception, this registry was reliant on regular patient follow-ups at the AKUH and KDSP. However, in Pakistan, the majority of patients do not have health insurance. This results in out-of-pocket payments for doctors’ appointments, testing, and procedures, which are often too taxing on the patient’s family. Furthermore, because of a scarcity of resources, there are few tertiary care centers where the needs of children with Down syndrome can be addressed. This results in further expenses as families need to travel far distances to receive care. Together, these problems create a large pool of patients who are lost to follow-up. In light of the COVID-19 pandemic, we further anticipate that patients will be reluctant to visit hospitals for interviews or regular checkups. We have used technology to bridge these deficits, with web-based consultations being carried out at a reduced fee at the KDSP with AKUH doctors to ensure that patients can be evaluated regularly from remote areas. Furthermore, the option for interviews via telephone calls or video calls is available, which we anticipate will make interviews more accessible to patients and their families. Concerns that arise from telephone interviews, such as a lack of facial cues regarding a patient or their family’s comfort level, are intended to be mitigated by the use of video call tools as well.

### Strengths

Being the first registry for patients with Down syndrome in Pakistan, the project offers insight into the demographics, morbidity, mortality, and economic burden for this patient cohort. This information is rarely available in LMICs, as cases are underreported and undertreated. These data can create an opportunity for region-specific interventions and changes that can address the specific problems faced by this population. Furthermore, through the web-based design, the registry offers a unique opportunity for physicians to receive real-time data regarding the patient’s HRQOL, development, and general health based on extensive questionnaires. Often, this information could be missed in a regular patient consultation because of time constraints. With this information, physicians can implement immediate interventions to reduce any difficulties or stressors experienced by the patient and their family, creating true patient-centered care.

### Conclusions

With the development of a Down syndrome registry, the opportunity to study Pakistan-specific trends in patients with Down syndrome presents itself. Given the vast differences in culture, social, and economic situations, discrepancies are expected between the quality of life of patients in LMICs and high-income countries. However, the assessment of HRQOL and patient-reported outcomes will allow us to see how this condition affects patients, how they and their families view their condition, and what their specific concerns are. This will allow the implementation of patient-centered care, a concept that is highly acclaimed yet often lacking in LMICs. This information will prove invaluable for implementation and influencing decisions for other patient populations with demographics similar to those seen in Pakistan. Although this study is currently limited to 2 centers in a major city of Pakistan, with the collection of data and initiation of this project, we are confident in the ability to procure funding soon to expand the project to regional, national, and eventually international levels.

## Abbreviations

AKUH: Aga Khan University Hospital
HRQOL: health-related quality of life
KDSP: Karachi Down Syndrome Program
LMIC: low- and middle-income country
PedsQL: Pediatric Quality of Life
